# Comparing the Hinge-Type Mobility of Natural and Designed Intermolecular Bi-disulfide Domains

**DOI:** 10.3389/fchem.2020.00025

**Published:** 2020-01-28

**Authors:** Philip Horx, Armin Geyer

**Affiliations:** Faculty of Organic Chemistry, Philipps-University, Marburg, Germany

**Keywords:** hinge-peptide, bi-disulfide, modeling, antibodies, metadynamic

## Abstract

A pair of intermolecular disulfide bonds connecting two protein domains restricts their relative mobility in a systematic way. The bi-disulfide hinge cannot rotate like a single intermolecular disulfide bond yet is less restrained than three or more intermolecular disulfides which restrict the relative motion to a minimum. The intermediate mobility of bi-disulfide linked domains is characterized by their dominating opening and closing modes comparable to the mechanics of a door hinge on the macroscopic scale. Here we compare the central hinge region of Immunoglobulin G1 (IgG1) which is highly conserved among different species, with a recently designed hinge-type motif CHWECRGCRLVC from our lab, that was successfully used for the dimerization of the IgG1/κ-ab CL4 monocolonal antibody (mab). The minimal length of these synthetic hinges comprises only 12 amino acids, rendering them ideal models for computational studies. Well-tempered metadynamics was performed to adequately describe the available conformational space defined by the different hinges. In spite of the differences in amino acid composition and ring sizes, there are characteristic similarities of designed and natural hinges like the dependent mobility of the individual strands of each hinge domain.

## Introduction

**Figure 1:**
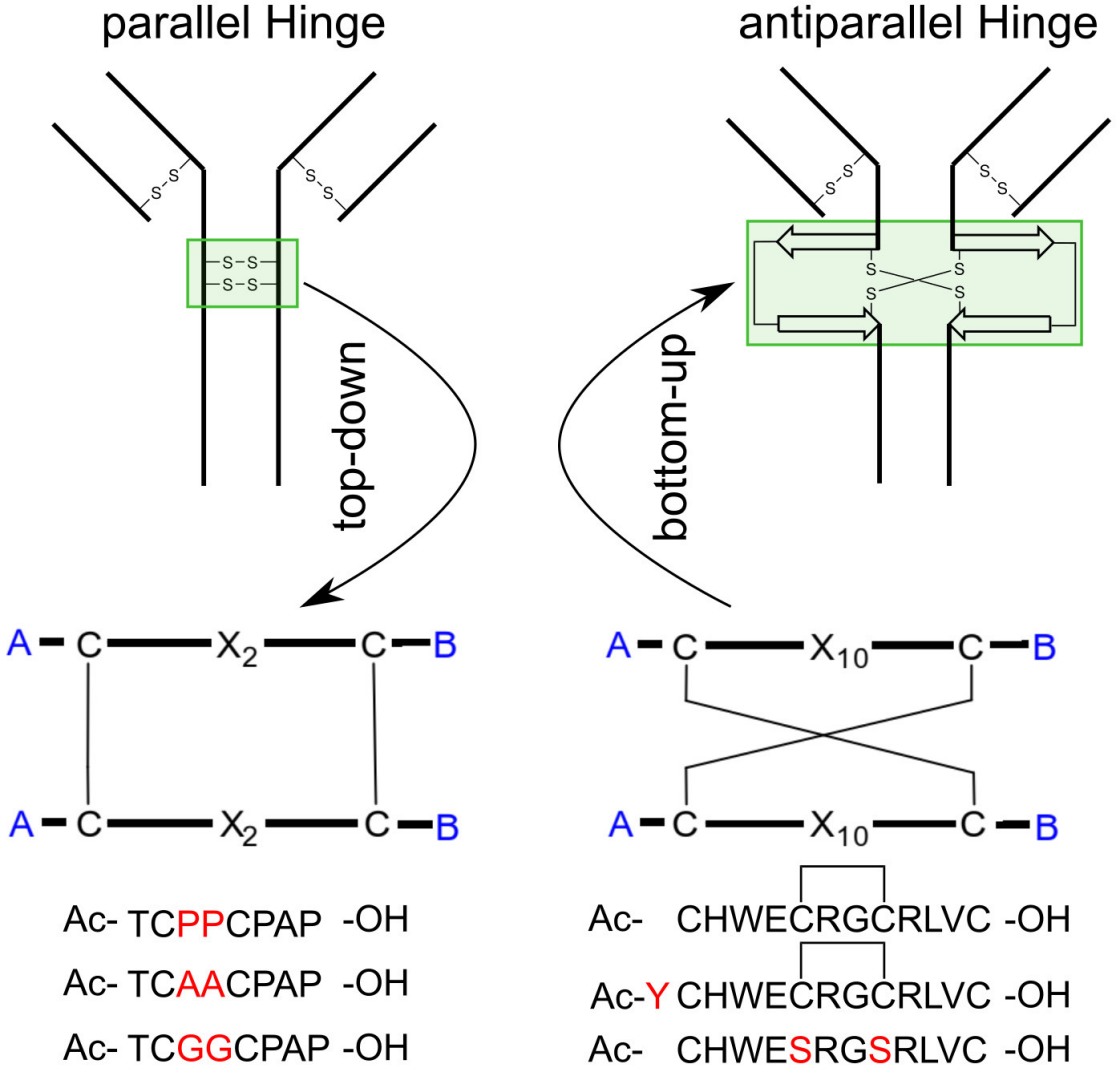
Applied methods and target peptides of this study. Using a top-down approach to identify the hinge-region and gain insight about the conformational dynamics. Transferring this knowledge to an antiparallel connected hinge peptide developed in our group. In future work, bottom-up methods can be applied, to design a IgG-derived antibody, containing an antiparallel hinge-domain.

The family of monoclonal IgG antibodies, which play a crucial role in the regulation of the human immune system, contain at least two intermolecular disulfide bonds as a highly conserved structure motif that form the only covalent links between the two identical halves of the Y-shaped IgG (Milstein, 1966; Frangione and Milstein, 1967; Pink and Milstein, 1967a,b). This central hinge region is flanked on both sides by the largely unstructured upper and lower hinge region (Vidarsson et al., 2014; Kim et al., 2016). The amino acid composition of the CXXC motif (Chivers et al., 1997) and the number of additional disulfide bonds which form the hinge domain, determines the categorization into the corresponding IgG subclasses (Schur, 1988). Both IgG1 and IgG4 contain the minimum of two intermolecular disulfide bonds, differing only by a P → S mutation in the central hinge CPPC (IgG1) and CPSC (IgG4). Already this single mutation leads to distinctly different tendencies of isoform formation for IgG1 and IgG4 antibodies (Angal et al., 1993; Bloom et al., 1997). The successful development of therapeutic monoclonal antibodies (mab) and mab-drug conjugates requires hinge regions with minimal tendency to isoform formation due to disulfide scrambling (Lowe et al., 2011; Wang et al., 2011; Pacholarz et al., 2016; Moritz and Stracke, 2017; Ashoor et al., 2018). The low activation barrier of disulfide exchange makes the thermodynamic stability of hinge folds the preferred optimization parameter to suppress disulfide exchange. According to this line of thought, we set out to characterize the hinge mobility by contemporary computational methods. The idea of synthesizing and modeling a single hinge peptide required the combined expertise of peptide synthesis and modeling groups in 1991 (Kessler et al., 1991). The present study uses enhanced sampling techniques to identify the dominating modes of natural and designed hinges, with the aim of identifying the structural characteristics of a thermodynamically stable hinge fold. The designed, yet DNA-encodable hinge peptides consist of a short 12mer sequence containing two or four cysteine residues which dimerize selectively to form the antiparallel hinge peptide (Schrimpf et al., 2017, 2018). NMR-based restraint modeling identified the opening and closing mode of the designed hinge, as well as a twisting motion of its two β-hairpin moieties (Horx et al., 2020, under review). Here, we expand our modeling strategy to more examples of designed and natural bi-disulfide domains with the aim of ranking similarities and differences of the individual hinge mobilities. We investigate the increase in mobility caused by the removal of the intramolecular disulfide bond in the designed hinge and we replace the proline residues with alanine or glycine in the natural IgG1 hinge region, respectively (Figure 1). The systems show clear differences in their flexibility although the overall hinge-type mobility of the disulfide clusters remained similar. To study this mobility, molecular dynamic simulation can serve as an ideal tool for investigations of dynamic behavior at the atomic level.

The major drawback of the standard methodology in MD-simulations is the inefficiency to sample rare events since the structure can be trapped in a local energy minimum (Chen et al., 2013; Bergonzo et al., 2014; Liao and Zhou, 2014). To overcome this issue, several enhanced sampling methods have been developed over the recent years (Bernardi et al., 2015). They can be loosely categorized into biased and unbiased methods. For the latter category one prominent example is temperature-based replica exchange MD simulation (TREMD) (Sugita and Okamoto, 1999; Kubitzki and de Groot, 2007) which achieves increased sampling by simulating numeral instances of the same structure (replicas) at different temperature and allows for transition between the replicas with a certain acceptance factor. One well-established enhanced sampling formalism using biased parameters is metadynamics (Laio and Parrinello, 2002). Hereby, the aim is to identify collective variables (CVs) which contain crucial collective movements and deposit a energy penalty on previously visited conformations during the simulation. This procedure compels the system to leave local minima and explore the conformational space more efficiently in less simulation time. As a beneficial side effect, the free energy profile of the investigated structure alongside the CV is generated and helps in the understanding of complex biological dynamics (Capelli et al., 2019; Fernández-Quintero et al., 2019; Kumar et al., 2019). The success of metadynamics methodology strongly depends on the identification of appropriate CVs since simulations with unsuitably chosen parameters do not converge and thus provide inaccurate results (Pfaendtner and Bonomi, 2015; Bonomi et al., 2016). To overcome this challenge, we used principal component analysis (PCA) to determine that the twisting of the disulfide clusters plays a key role in the overall dynamic of hinge-type connections. To evaluate the similarities between antiparallel and parallel hinge-type connections, the described modeling protocol was used to reveal a coupled dynamic of the disulfide cluster depending on modification inside and outside of the hinge core. We hope that the knowledge about the dynamics of parallel and antiparallel hinges will enable us to design unique antiparallel orientated antibodies with specific structure-activity relationships.

## Materials and Methods

### NMR Structure Determination

The starting structures for the MD-Simulations were generated using the Xplor-NIH suite of programs (Schwieters et al., 2003). Distance constraints for the hinge peptide derivatives were extracted from NOESY spectra with a mixing time of 300 ms. The cross-peaks were organized according to their intensities as weak, medium or strong (2.1–3.1Å). Due to the flexibility of the hinge peptide as observed in NMR the focus was shifted on a small set of well-defined distance constraint which describe the system sufficiently. This resulted in a total of 23 individual restraints for one chain (46 counting the second strand). Further assessment of coupling constants and cross-peaks revealed preferred side chain orientations which were included as a dihedral-angle-constraint during the calculation protocol. Since the system exhibits a higher flexibility mitigation of the constraints was pursued to achieve a higher sampling of possible starting structures. The IgG-antibody hinge regions distance constraints were taken from the literature (Kessler et al., 1991). This data set yielded a structure assemble with multiple structure with no NOE- and dihedral-violations above 0.5 Å. The calculations started from an extended conformation using the torsion angle dynamics simulated annealing protocol written by Stein and coworkers (Stein et al., 1997). The starting structure, acetylated at the N-terminus, started with all disulfide bonds already in place. The C-terminus was deprotonated, to mimic the experimental conditions under which the hinge peptide was measured. The system was heated to 3,500 K and cooled down in 12.5 K steps. The eefx2 implicit force field was chosen since it displays higher accuracy and quality of the calculated structure in comparison to the native state (Tian et al., 2017). The calculations were performed with 5,000 structures while the 10 most stable conformations were extracted. The structures with the lowest energy were chosen as the starting structure for all further molecular dynamics simulations. Structural representation were generated using the VMD graphics suite (Humphrey et al., 1996) or pymol (Pymol)^1^. The modified annealing protocol is contained in the Supplementary Material.

### MD Simulations

All simulations were performed using the GROMACS 2018.4 (Berendsen et al., 1995; Abraham et al., 2015; Páll et al., 2015) suite. For input preparation the lowest energy structures originating from the NMR calculations was chosen and processed using the pdb2gmx program. The peptides were solvated in respective to system size with 3000–6000 TIP3P water molecules in a dodecahedron box. A salt concentration of 0.15M was added to neutralize the system. A recent study conducted in our group showed that the OPLSAA/M force field (Robertson et al., 2015) is a suitable choice for describing the dynamics of hinge peptides. Energy minimization was performed for either 500000 steps or until the maximum force reached a value below 50 kJ/mol/nm using steepest-descent algorithm to remove steric clashes between the peptide and solvent. The next step included a 10 ns long equilibration to allow the solvent to fully surround the peptide. The first equilibration phase was conducted for 1 ns under a NVT ensemble at 300 K using the modified Berendsen thermostat v-rescale (Bussi et al., 2007) with a coupling time step of 0.1 ps to stabilize the temperature of the system followed by an 2 ns long NPT equilibration to stabilize the pressure using the Berendsen barostat with a coupling time step of 2.0 ps. Particle-Mesh Ewald for the treatment of electrostatic interactions was employed with a short-range cut off of 1.0 nm. For the final unrestrained production run the Parrinello-Rahman barostat (Parrinello and Rahman, 1980, 1981) was used and the system subjected to a 2200 ns long run at 300K. Root-Mean-Square Deviation (RMSD) was measured using implemented tools in the GROMACS package. To analyze the functionally most dominant motions Principal Component Analysis was performed on the whole trajectory of the classical MD-Simulation with more detailed information present in the supporting information. Defining the opening angle of the hinge peptide the geometric center between the alpha carbon atoms of cysteine/serine 5 and 8 and the center of the four sulfur atoms of cysteine 1 and 12 were taken for the hinge peptide. The structural stability of the hinge core was measured by an dihedral angle composed of the alpha atoms of the cysteine residues involved in the formation. For the hinge peptides the sequence was C1-C12′-C1′-C12 and C3-C3′-C6′-C6 respectively.

### Well-Tempered Metadynamics

For enhanced sampling simulation Gromacs 2018.4 patched with plumed 2.4 (Tribello et al., 2014). was used to increase sampling of conformational space. To determine a defined set of biased parameters (CV) principal component analysis was performed and yielded the twisting of the β-hairpin-moieties as a suitable parameter for the hinge peptide. The IgG-derived hinge peptide was biased using the dihedral angle of the disulfide cluster. For the well-tempered metadynamics a Gaussian with a height of 0.175 kJ/mol and a bias factor of 8 was deposited every 1 ps for the hinge peptides. These dihedral angle was biased with 1.2 kJ/mol and 10 since the original values led to an unfolding of the secondary structure. The Gaussian width was to 0.05 or 0.35 respectively. These settings were chosen to allow the sampling of the conformational space in a reasonable amount of simulation time while still allowed to visit smaller energy basins. For the free energy surface calculation a weighted histogram approach along the opening angle and tryptophan-tryptophan distance was used. The bin size was set 425 points.

## Results

### NMR-Structures of Parallel and Antiparallel Hinge-Type Connections

**Figure 2:**
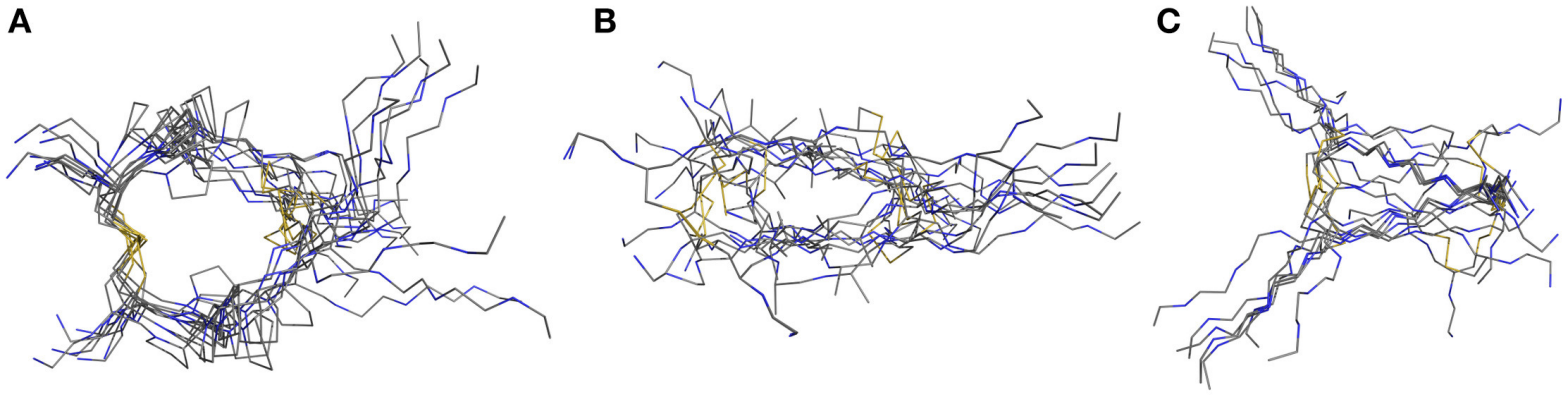
NMR-derived structure ensemble of the IgG hinge-domain derived peptides. The native structure [labeled Pro-IgG **(A)**] shows a well-defined core stabilized by proline residues. The alanine **(B)** and glycine **(C)** derivatives display a higher variance in their core-region.

The in 1991 investigated hinge-domain of the IgG antibody serves as interesting target for analysis of disulfide-rich peptides (Kessler et al., 1991). At that time, the emphasis on the hinge domain, which is responsible for the dimerization of heavy chains, has been an essential part of investigations into the mobility and activity of antibodies (Toyofuku et al., 1992; Kim et al., 1995). Following this mindset we used the hinge-domain sequence of IgG1 (TCPPCPAP) for the mobility study of parallel hinge-type connections. Initial analysis was performed by NMR-derived structure-ensembles generation using the simulated annealing protocol described in the materials and methods section. To investigate the importance of stability of the disulfide core to the overall hinge-type dynamic modifications by replacing P3 and P4 with alanine or glycine residues were accomplished. NMR-structure calculation revealed with the used protocol large differences in the lowest-energy ensembles due to the larger flexibility of the system (Figure 2). The proline containing sequence (further on named Pro-IgG) showed the lowest RMSD values after alignment of the NMR-structures. This feature can't be retained for Ala-IgG and Gly-IgG which differ substantially in their ensemble due to reduced stability in the disulfide core region. Even though glycine residues retain more flexibility their NMR structure is more defined with the chains following the core-region clearly aligned.

**Figure 3:**
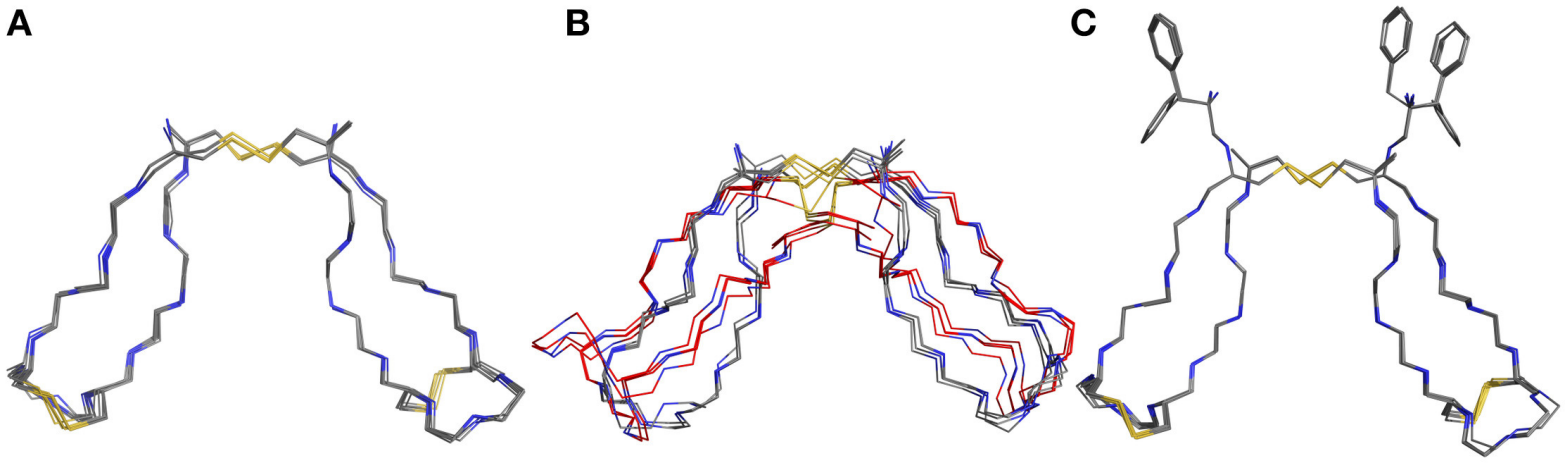
NMR ensemble of hinge peptide derivatives. The native peptide **(A)** with four disulfides shows a small deviation in their NMR-structures. The replacement of the intramolecular disulfide ensemble with serine residues **(B)** shows stronger variance due to lower stability. An elongation with tyrosine **(C)** leads to no significant change in structure variance.

For assessment of the dynamics of antiparallel dimerized hinge-type connections we chose a DNA-encodable 12 mer sequence (CHWECRGCRLVC) that was recently established by our group and is called hinge peptide (Schrimpf et al., 2017). Initial structural analysis again was elucidated by NMR-derived structure generation. To further increase the understanding of hinge-type disulfide-rich peptides dynamics we modified the mobility of the β-hairpin and disulfide core region. Upon removal of internal disulfide (C5/C8) we observed higher variance in the lowest energy structures obtained after NMR-derived structure calculation. This behavior is expected since the disulfide present in the parent structure removes certain degrees of freedom and thus renders a more concise structure ensemble. Modification of the disulfide cluster by extending the sequence by one tyrosine residue shows a smaller influence on the overall structure-ensemble. Even though there are some disparities in the orientation of the tyrosine or tryptophan residue the overall motif stays consistent and shows the characteristic formation of the antiparallel β-sheet which is restricted in the β-turn region due to the intramolecular disulfide. The twisting of the β-hairpins remains almost identical for all derivatives and can therefore be identified as a structural key element of the antiparallel hinge as seen in Figure 3.

### Evaluation of Structural Stability Using MD Simulation

**Figure 4:**
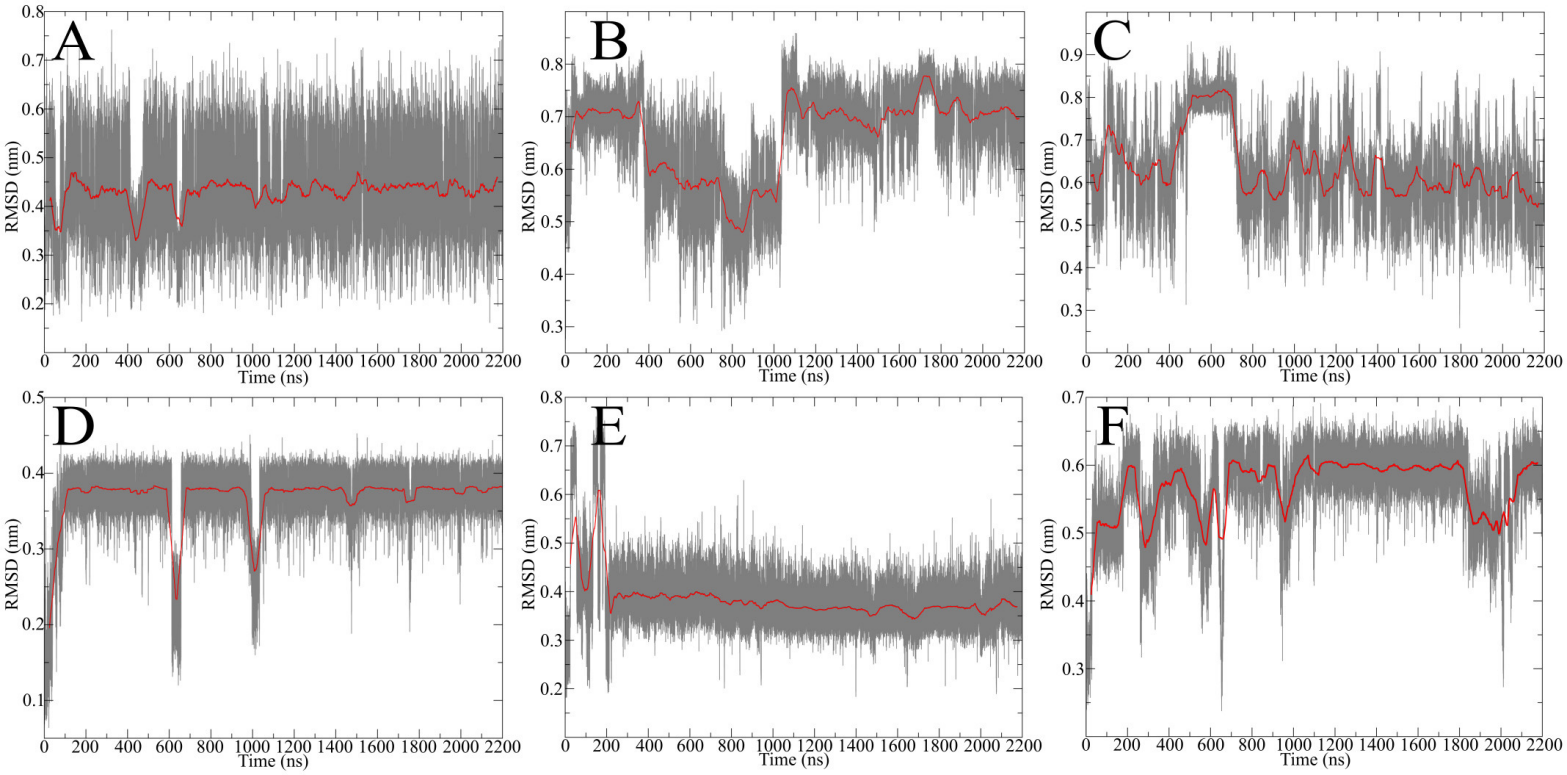
RMSD evolution of hinge-derivatives. The black faded line display the progression over time with an average over 10 ns indicated by the red line. The Pro-IgG **(A)** shows only small changes in RMSD in contrast the alanine and glycine derivatives **(B,C)** both show larger and more frequent changes. Similar behavior can be observed for the hinge peptide and derivatives. The native sequence **(D)** shows a stable RMSD of 0.38 nm with rare changes. Removing the intramolecular disulfide has no immediate effect on the RMSD **(E)**, while elongation by tyrosine **(F)** leads to more frequent changes during the simulation.

After obtaining starting structure for the disulfide rich peptides we wanted to investigate their dynamic behavior in solution at an atomic level of detail. Therefore, standard MD simulation was selected as the method of choice to obtain initial results about their dynamical properties. We recently established guidelines for the description of hinge-type connections and identified a workflow for obtaining information about conformational dynamics in a concise manner (Horx et al., 2020, under review). Root-mean-square deviation (RMSD) evaluation is able to uncover differences in structural stability over the course of the simulation and therefore serves as an ideal prefatory parameter to evaluate hinge dynamics. At first the IgG derived hinges-domains were examined in Figure 4 revealing that Pro-IgG only shows small dibs in the RMSD and stays at 0.45 nm. The change in the core region to alanine leads to massive change in RMSD fluctuation with a massive jump in RMSD as it drops from 0.7 to 0.5 nm and back. This behavior is maintained in the Gly-IgG with a large jump at 500 ns. Afterwards the system performs larger changes more frequently than the other two derivatives. This is due to the increased flexibility originating from the amino acid composition. The native hinge peptide exhibits a similar behavior in comparison to Pro-IgG, namely the RMSD quickly stabilizes at 0.38 nm and only infrequent changes are taking place in the conformational composition. Surprisingly substituting the intramolecular disulfide bond with two serine residues does not in fact yield in a higher fluctuation of RMSD as observed in the NMR-structure calculations but converges after initial equilibration to the RMSD as the native sequence. On the contrary, the derivative elongated by tyrosine (Figure 4F) shows more frequent transitions even after 1,000 ns simulation time and reaches a higher RMSD overall at 0.6 nm. The insight of structural stability obtained through classical MD simulations reveals that both native sequences (Pro-IgG and hinge peptide) observe similar stability with only small changes in conformation after initial equilibration. Substitution of the core-region negates the stabilizing rigidity which is especially apparent in the IgG-derivatives since Ala-IgG and Gly-IgG both exhibit frequent structural fluctuations. The hinge peptide tolerates substantial structural change since the removal of the intramolecular disulfide bond has little to no effect on the RMSD value. Steric interactions introduced by tyrosine in the disulfide core region has a more obvious effect which is reflected in the frequent RMSD transitions.

### Identifying Hidden Dynamics Using Principal Component Analysis

**Figure 5:**
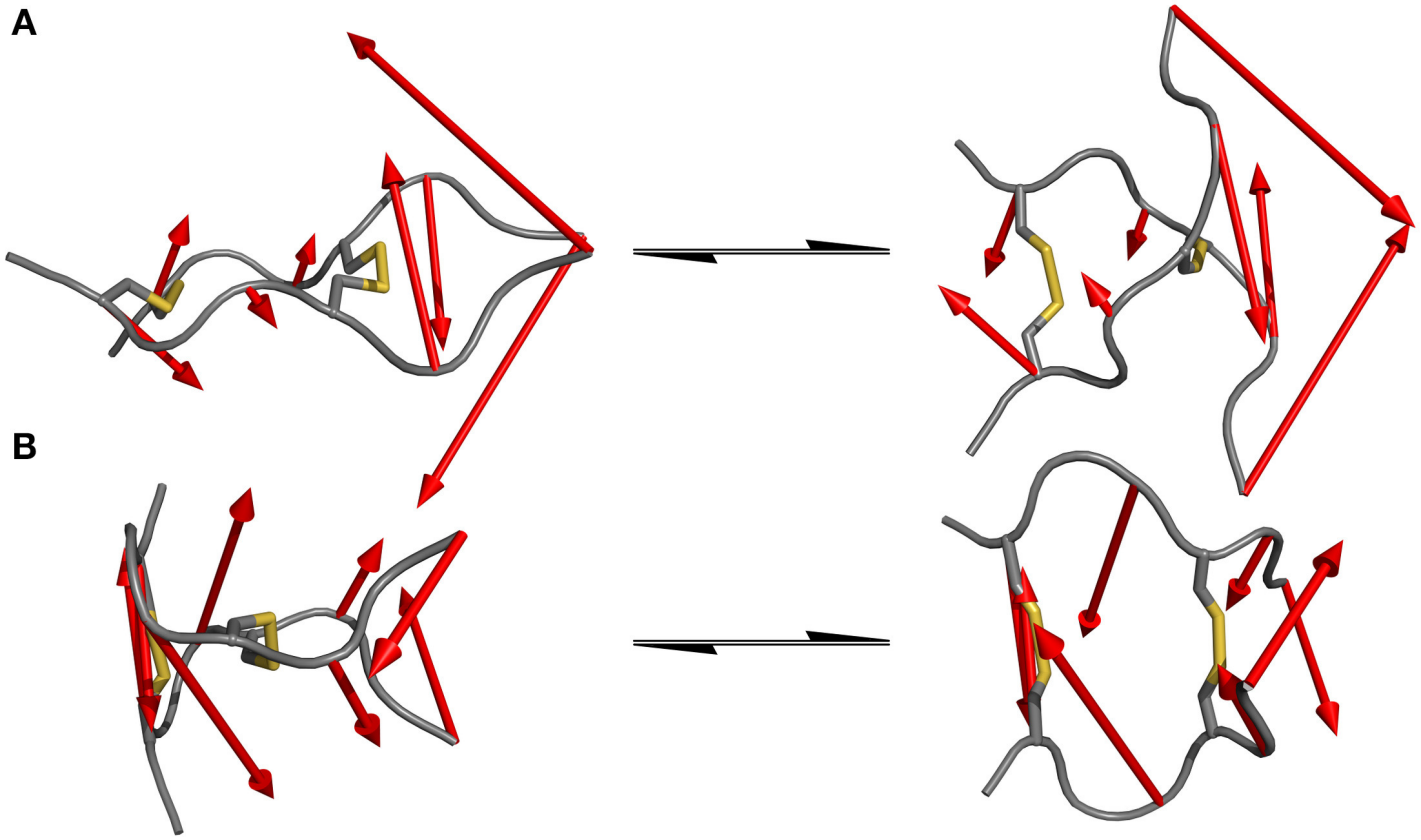
Dominating motions revealed by PCA of IgG hinge derivatives. The opposite translation of the C-termini out of the peptide plane is identified as the most prominent movement in the Pro-IgG hinge **(A)** while the second most important motion is represented as the opening and closing of the core region **(B)**.

Since standard MD simulation is often unable to sample the conformational space of a given system sufficiently, enhanced sampling methods were used to overcome this issue (Bernardi et al., 2015; Yang et al., 2019). Well-tempered metadynamics serves as an ideal tool to increase sampling without additional significant computational cost. Selection of the biased parameter necessary for metadynamics is the critical step for efficient improvement in sampling. As has been highlighted principal component analysis (PCA) serves as an optimal technique for the identification of hidden dominant motion (Maisuradze et al., 2009). These motions can then be used as a CV to perform biased simulation and gather information about the energy barriers separating those conformers. After applying PCA to whole data set of unbiased MD simulation for the IgG derivatives, the native sequence Pro-IgG exhibits one distinguishable significant movement. The swinging past each other of the longer chains after the second disulfide (P6-P8) accounts for 54% of the overall motions. This movement is still present in Ala-IgG and Gly-IgG but other motions such as opening and closing by the disulfide region play a greater role due to the reduced stability of the hinge core. It is furthermore more difficult to identify hidden dominating motions since increased flexibility leads to multiple changes at different conformational positions. This reduced stability is due to the increased conformational mobility in the alanine and glycine residues located inside the disulfide core. To visualize those principal components the starting and end conformations of one separate motion were taken and the vector between the alpha carbon atoms visualized by an arrow seen in Figure 5.

**Figure 6:**
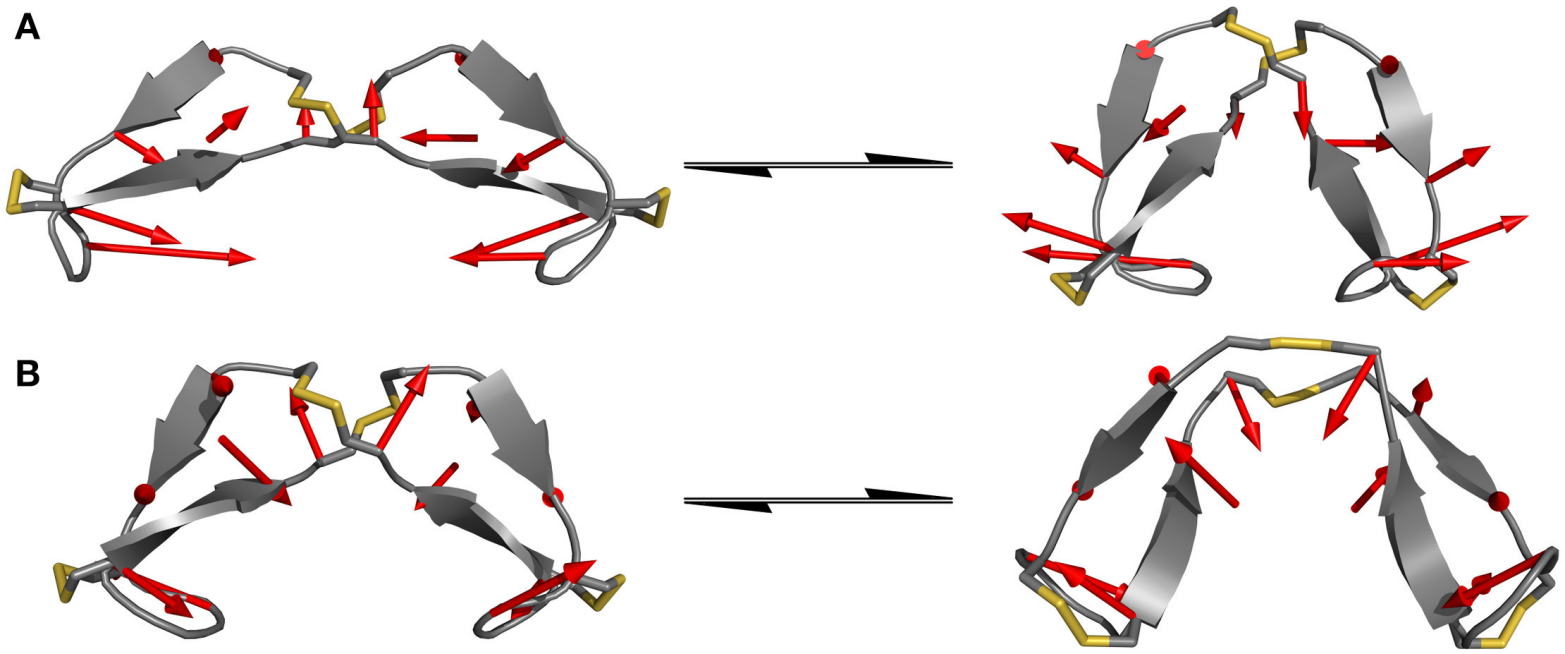
Dominating modes of the hinge peptide identified by PCA. Row **(A)** shows the opening and closing motion with little to no twist of the β-hairpin moieties, while row **(B)** is indicating the twisting of the β-hairpin-moieties, whereas the opening angle remains largely the same. The red arrows indicate the starting and endpoint between the two structures.

Since the hinge peptide exhibits as more rigid structure overall, applying PCA yielded two clearly identifiable motions, namely twisting of the β-hairpin moieties as the main dominating motion which accounts for 55% of the overall motions and opening and closing of the hinge with only 11%. This twisting of the β-hairpin motifs could be described as a formation of a right-handed double-helical structure with isoenergetic bending of hydrogen-bonds which retains the symmetry and thus stabilizes the overall structure (Salemme, 1982). These two motions are visualized in Figure 6. Those values are in the same magnitude for the other derivatives. The serine containing hinge peptide shows an increase in opening and closing motion to 21% while maintaining the twisting motion at 52%. Elongation of the chain by tyrosine reduces the impact of the opening and closing motion to 9%, with the proportion of twisting increasing slightly to 57%. Although the difference is minimal, this may indicate a shift of the conformation in the more open region since steric repulsion of the elongated chain energetically deprecates the closed conformation. The sequences and their respective movements are summarized in Table 1.

**Table 1:** Percentage of the dominating modes of different hinge peptide derivatives.

**Sequence**	**Amount of twisting motion (%)**	**Amount of opening and closing motion (%)**
Hinge peptide	55	11
Serine hinge peptide	52	21
Tyrosine hinge peptide	57	9

**Figure 7:**
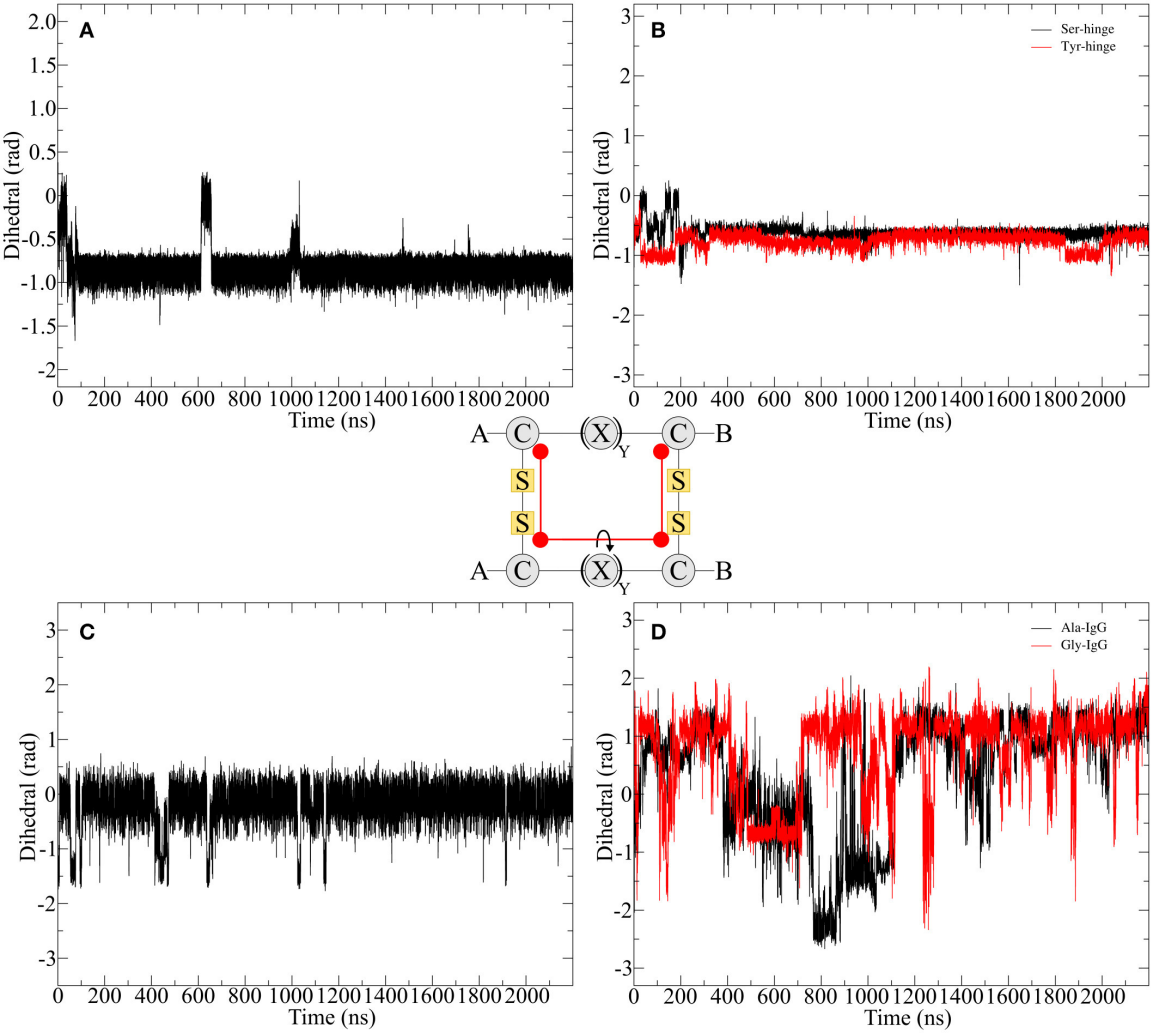
Disulfide core dihedral angle evolution during the simulation of different hinge type connections. The native hinge peptide exhibits a slight twist at −1 rad **(A)** which is comparable to the derivatives **(B)** with slightly smaller dihedral angle. A similar pattern can be observed for Pro-IgG **(C)**, whereby the torsion is almost 0 rad indicating a parallel orientation of the chains. Upon modification huge fluctuations with torsion angles ranging from 1.5 to −2 rad are observed **(D)**.

In order to compare the hinge dynamics among different hinges, neither the twisting of the β-hairpin moieties nor the swinging by of the chains serve as an ideal parameter since structural differences are too severe. The closest similarities can be found in the disulfide cluster of the hinge which brings it into focus for subsequent investigations. Twisting of the β-hairpin residues and swinging of the chains toward each other also results in a twisting of the disulfide cluster, which can be effectively described by a torsion angle. The progression of time for the torsion angle spanned by the alpha carbon atoms of the cysteine residues is shown for all derivatives in Figure 7. Pro-IgG displays a fluctuation pattern which largely correlates to the RMSD-pattern. The disulfide core generally is almost planar (at 0 rad) but exhibits infrequent jumps to a more twisted conformation (−1.5 rad). The transition of the core region to more flexible alanine and glycine residues leads to massive increase in variance of the dihedral angle. There is no outstanding angle observable and large changes are happening more frequently hinting on a flexible structure. This claim is further supported by changes of up to −3 rad. As a common theme similarities are apparent between the two native sequences, as the hinge peptide displays a very stable slight twisting of the disulfide-region (−1 rad) only interrupted during equilibration phase and a small fluctuation at 600 ns. The serine and tyrosine derivatives behave in a comparable way, showing some twisting on average (−0.6 rad), but smaller than the native hinge. This indicates that the slightly twisted structure is the most prominent during the simulation.

### Enhancing the Occurrence of Rare Transitions—Well-Tempered Metadynamics

**Figure 8:**
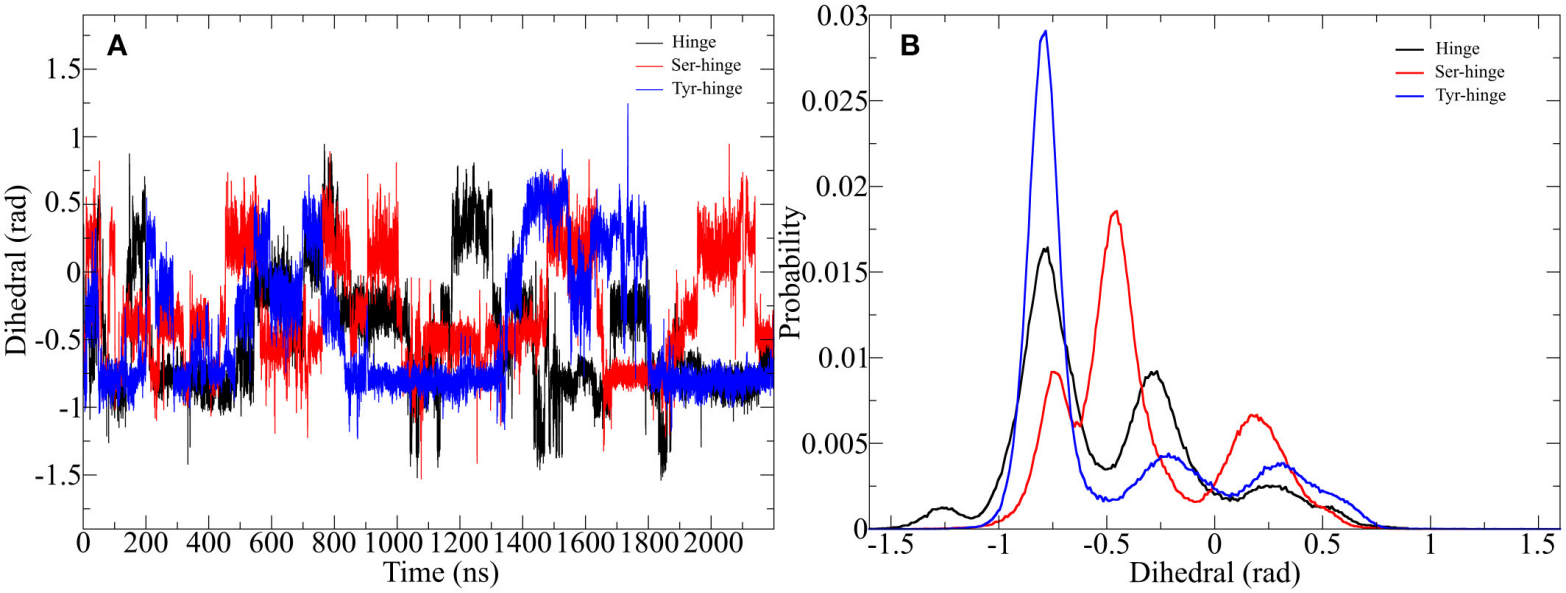
Impact of metadynamics simulation on the twisting of the disulfide cluster in the designed hinge peptide. **(A)** Depicts the evolution of the disulfide cluster dihedral angle during the simulation. The torsion changes rapidly during the simulation time frame but prefers a slightly twisted to twisted conformation. **(B)** Shows a histogram depiction of the derivatives, highlighting the different effects of residue substitution.

Figure 7 shows that the structural changes are of low frequency and in order to better understand those rare transitions, a variety of enhanced sampling methods have been developed. As mentioned above one of those methods is called metadynamics where a history-dependent potential is built as sum of Gaussian functions and added to a specific parameter called CV to compel the system to escape local minima. The addition to the well-tempered metadynamics allows the height of the Gaussian potential to decrease during the simulation which leads to a smoother conversion of the free energy landscape dependent on the CV (Barducci et al., 2008). A good estimation for the CV can be provided by PCA. Utilizing the findings from the previous chapter the twisting of the β-hairpin-moieties could isolated as an ideal parameter for enhancing transitions between the opened and closed state of the hinge. Therefore, we performed well-tempered metadynamics simulations for all hinge peptide derivatives and evaluated the evolution of the disulfide core twist as the comparable parameter between hinge-type connections in Figure 8.

The adaptive bias on the β-hairpin twisting clearly exercises a strong influence on the orientation of the disulfide core. Every hinge peptide derivative shows transitions to different angles at a high frequency. The dihedral angle switches from −1 to 0.5 rad remaining at a parallel orientation for a limited amount of time. While all three derivatives exhibit a similar pattern the histogram projection of those angles highlights the differences. The native hinge peptide shows the highest probability at −0.8 rad, while a smaller fraction of the conformations still occupies −0.3 rad. After substitution of the intramolecular disulfide bond the probability pattern changes substantially. Although −0.8 rad is again populated by a great number of structures, the most populated dihedral angle is −0.5 rad. Additionally, the increased flexibility of the β-hairpin allows the hinge core to adopt a different orientation at 0.2 rad to a significant extent. Elongation of the peptide sequence by tyrosine, on the other hand, leads to a restriction of the cores' mobility, whereby almost exclusively a twist of −0.8 rad can be observed with small category at −0.25 and 0.25 rad respectively. By using enhanced sampling techniques we were able to describe the extent of twisting of antiparallel hinges. Even through the energetic penalties of local minima, the antiparallel hinge peptide core was not able to reach the extent of twisting of IgG-hinge derivatives.

**Figure 9:**
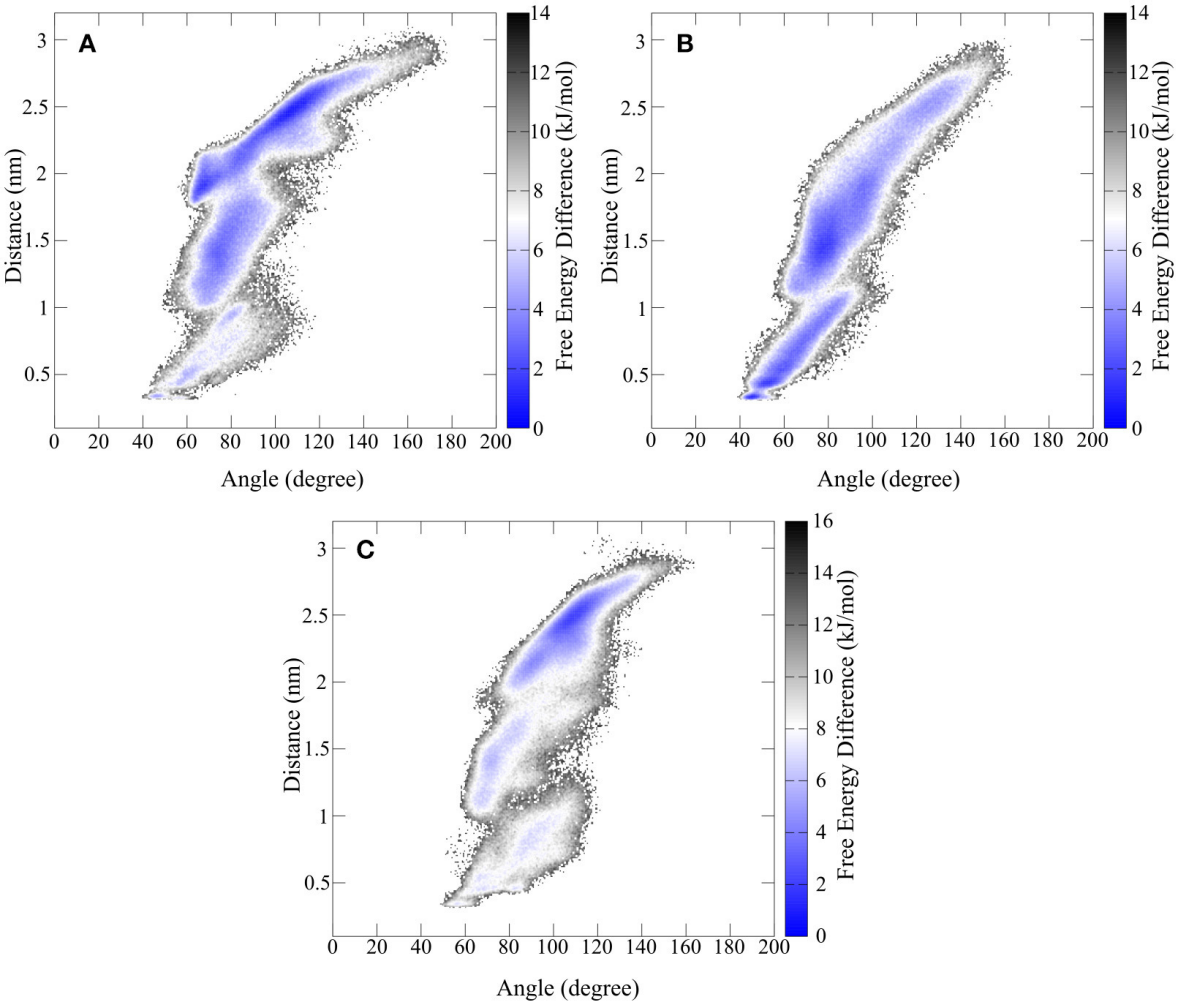
Free energy representation of the conformational space described by the twisting of the β-hairpin moieties and opening and closing of hinge motif. **(A)** Shows the FES of the synthetic hinge peptide with the lowest energy basin located at the extended twisted conformation. The lowest energy basin for the hinge peptide containing the serine residues **(B)** is shifted toward the closed untwisted conformation. **(C)** Highlights the narrowed FES occupied by the tyrosine elongated derivative.

Since the hinge peptide shows a twisting as well as the opening and closing of the β-hairpin motifs to each other as main motions, the description of the conformational mobility would be insufficient using only one variable. Therefore, free energy calculations were performed using a weighted histogram approach using the two dominant motions to characterize the conformational space as shown in Figure 9.

The overall extent and shape of the conformational area occupied by the hinge peptide derivatives shows large similarities. Opening angles from 40 to 140° are visited regularly although differences can be found in the occupancy of those areas. The removal of the intramolecular disulfide compels the hinge to adopt a closed structure (40 to 70°) more frequently than the restricted derivative. Elongation of the sequence by tyrosine has the opposite effect as the structure is more constraint in the larger opening angle areas. As we have shown the effect on the transposition of the β-hairpin motifs can be measured as a distance between two tryptophan residues. The same trend as with the opening angle can also be observed with the twisting. While the derivatives with intramolecular disulfides tend to favor larger distances and thus greater twisting, the serine derivative prefers small distances and thus an almost parallel orientation. In particular a small confined area of 43° and 0.3 nm describes the most compact structure. The most common structure for the native hinge peptide and tyrosine derivative is located at 2.5 nm twisting and 110° opening angle.

**Figure 10:**
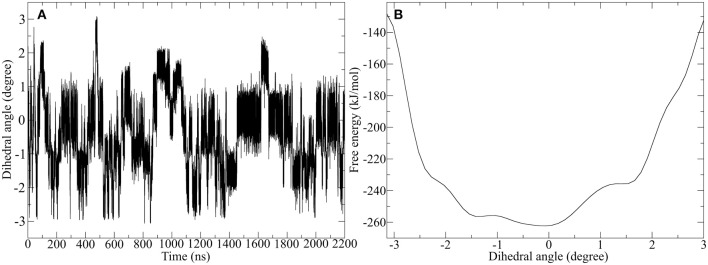
Well-tempered metadynamics of Pro-IgG hinge by biasing the dihedral angle in the hinge core. **(A)** Shows the evolution of the angle during the simulation and highlights a frequent switching between different levels of twisting. **(B)** Depicts the free energy evaluation of the twisting dihedral.

Since the Ala-IgG and Gly-IgG derivatives show already a highly fluctuation of the dihedral angle in the disulfide core, only the Pro-IgG sequence was subjected to metadynamics simulation. The hinge core torsion was used as the only CV to enhance the conformational sampling as shown in Figure 10.

In contrast to standard MD simulation, where the angle remains constant at −0.5 rad (Figure 7) with only occasional changes, the well-tempered metadynamics simulation is able to push the system out of the local minima and thus results in a significant variation of the torsion. In addition to stronger maximum and minimum twisting, the frequency of these alterations is also considerably higher. One additional advantage of well-tempered metadynamics is that after the simulation has converged an energy profile can be easily obtained alongside the CV. This profile demonstrates that the energetically most favorable angle is 0 rad, but due to low energy barriers the area from −1.5 to 0.5 rad can be readily explored at room temperature. Even though the hinge peptide designed in our lab differs in structural relevant functions key motions are present in both classes. The opening and closing of the chains between the intermolecular cysteine residues is only clearly present in the hinge peptide. While the IgG-hinge still exhibits this type of motion to a small percent the larger part is represented by a more complex dynamic behavior which is highly dependent on the twisting of the disulfide cluster.

## Discussion

Utilizing a top-down approach, we concentrated our investigations on the hinge-region of the human immunoglobulin G1 antibody, the heavy chains of which are linked by two disulfide bonds and thus exhibit a directed dynamic. Previous studies have indicated that the hinge-core of IgG1 adopts a stable polyproline II double helix and thus the internal flexibility is influenced by the chains outside of the core (Kessler et al., 1991). PCA on the classical MD simulation data set further supports this claim as the main motion is a swinging by of the chains following the core region. To gain further insight into this dynamical behavior and the role of core stability, we modified the core residues to gain the alanine and glycine derivatives. As expected, both derivatives exhibit different conformational stability by showing frequent jumps in the RMSD progression. This increase in structural flexibility causes a diversification of the dynamical space as confirmed by PCA as the major motion now contains a swinging of the N-terminal chains in conjunction with compression of the hinge core. We furthermore wanted to compare the parallel orientated hinge with an antiparallel connected hinge recently established in our group. The 12 amino acid, four cysteine containing sequence oxidizes selectively to form the tetra-disulfide antiparallel hinge. RMSD investigations show a stable RMSD with small infrequent jumps similar to the parallel IgG1 hinge. In comparison to the parallel hinge core we also included modifications of the core region by removal of the stabilizing intramolecular disulfide and elongation by a tyrosine residue. The impact of these modifications on stability evaluated by RMSD is not as pronounced as in the IgG1 derivatives. It has been noted that the elongation with tyrosine has a greater influence on destabilizing the core region as it allows more frequent changes in RMSD during the simulation. Reveal of the hidden dynamics was furthermore evaluated using PCA and showed that the twisting of the β-hairpin moieties in combination with opening and closing of the hinge angle accounts for over 70% of the complex dynamics. The extent of the individual movements was influenced to varying degrees by the modifications. Increasing the flexibility of the β-hairpin enhances the proportion of opening and closing movements of the hinge angle, while the extension of the chain causes a focalization on the twist. In order to achieve enhanced comparability between the parallel and antiparallel categories, we sought a parameter that could be applied to both categories, despite the structural differences. The twisting of the hinge core could be identified as an ideal parameter to describe conformational stability. Progression of time showed again large similarities in core stability for both native sequences. The major difference lies actual amount of twisting, whereas the parallel hinge adopts an almost parallel alignment of the chains, the antiparallel hinge peptide maintains a slight twist of −1 rad. This stability is maintained for both synthetic hinge peptide derivatives as depicted in Figure 7B. Large differences can be observed for the core stability in the Ala-IgG and Gly-IgG derivatives. The disulfide core angle changes frequently during the simulation with fluctuations up to 3 rad. One issue with using MD simulation for elucidation of dynamical properties is the incapability to overcome local energy barriers and sample the conformational space sufficiently. We utilized the well-tempered metadynamics method, since numerous examples in the literature have shown that by employing accurate CVs the, precise description of the conformational space is feasible (Limongelli et al., 2013). The twisting of the β-hairpin motifs to each was identified by PCA and was employed as the CV for the hinge peptide. Increase in sampling was observed for all synthetic hinge peptide derivatives as the core twisting frequently changes up to 1.5 rad, yet differences in the torsion distribution could be measured. The native hinge peptide favors two conformations, −0.75 and −0.25 rad. Mutation of the intramolecular disulfide by serine yields in a twist of −0.5 rad and possible inversion to 0.25 rad. The opposite effect could be observed for the tyrosine elongation as it narrows the distribution to −0.75 rad. To get a better understanding about the conformational flexibility, a free energy surface was projected on the two dominating modes, which further supports the effects of modification in the hinge peptide. The native sequence is able to adopt multiple conformations due to low energy barriers separating the structures. A shift toward the closed and untwisted conformation is observed by the Ser-hinge peptide while again tyrosine elongations leads to preference of the extended and twisted structure. Since the dihedral angle of the disulfide core for the derivatives Ala-IgG and Gly-IgG already changes frequently, we have determined to subject only Pro-IgG to a well-tempered metadynamics simulation. This time we biased the disulfide core torsion itself to evaluate the amount of increased sampling and furthermore obtain a free energy curve. The dihedral angle undergoes large fluctuations between different states of twisting up to 3 or −3 rad with frequent transitions between the those conformations. Evaluation of the free energy profile reveals that the angle at 0 rad could be identified as the lowest energy conformation but energy barriers around 20–30 kJ/mol allow the system to leave the global minimum infrequently and reach other local minima at −2 and 1.5 rad. This further supports the assumption that the CPPC hinge core is rigidified yet able to cross those barriers depending on the constitution of the upper and lower hinge domain. Comparison of both hinge peptides reveals that similarities lie in the dynamic of the disulfide core yet the antiparallel hinge peptide shows less flexibility and a narrowed dominant motions in the opening and closing of the hinge with accompanying twisting of the β-hairpin motifs. This insight could be used for the design of antiparallel hinge containing mabs. Intermolecular bi-disulfide requires significant thermodynamic stabilization in order to compensate the entropy loss. Both investigated hinge-types show preferred intermolecular disulfide bonding whereby the peptide with the highest cysteine content (CHWECRGCRLVC) exhibiting the highest dimerization selectivity. In spite of this thermodynamically preferred oxidation pathway, it retains a hinge-type mobility characterized by the hidden dominant motions, identified by PCA and the free energy surface obtained by well-tempered metadynamics simulations. The design of isoform free mabs will profit from the development of Cys-rich peptides with high pairing specificity and sufficient freedom of movement under the structural restraints of attached immunoglobulin domains.

## Data Availability Statement

The datasets generated for this study can be found in the OSF depository https://osf.io/jqz3k/?view_only=83558bc693b84753a0eb20d7f7caf0ed.

## Author Contributions

The experiments were designed by PH and AG. PH performed the simulations and analyzed the data. The manuscript was written by PH and AG.

### Conflict of Interest

The authors declare that the research was conducted in the absence of any commercial or financial relationships that could be construed as a potential conflict of interest.

## Footnotes

^1^The PyMOL Molecular Graphics System, Version 2.0 Schrödinger, LLC.

## Supplementary Material

Further Information about the simulation procedures and additional figures can be found in the supporting information. Additionally, short videos of the main motions of the different hinge peptides can be found in the supporting information.

Video 1 This video shows the projection of the trajectory of the designed antiparallel hinge peptide on the first (and most dominant) eigenvector. This results in a twisting motion of the hairpin to another.

Video 2 This video depicts the projection of the parallel hinge peptide's trajectory on the first (and mot dominant) eigenvector. This results in a swinging past of one another of the longer chains.

Video 3 This video depicts the projection of the designed antiparallel hinge peptide's trajectory on the second eigenvector. This highlights the opening and closing motion of the hinge.
