# Supplementary material for: Comparing the Hinge-Type Mobility of Natural and Designed Intermolecular Bi-disulfide Domains
Source: Front Chem. 2020 Jan 28;8:25. doi: 10.3389/fchem.2020.00025 (PMC6997481; doi:10.3389/fchem.2020.00025)
Supplement: Supplementary file 4 [file Data_Sheet_1.PDF]

Supplementary Material for Comparing the hinge-type mobility of natural and designed intermolecular bi-disulfide domains.

Philip Horx, Armin Geyer

Principal Component Analysis (PCA)

We performed PCA on the whole data set of the classical molecular dynamics simulations to get an understanding of the most functional modes defining the dynamic of the hinge-type derivatives. First the positions of the peptide atoms were aligned followed by the calculation and diagonalization of the covariance matrix. We opted for depiction of the first 10 eigenvalues since they account between 70-80% of the overall motions of the peptide. Both native sequences (hinge-peptide and Pro-IgG) show one largely dominating Eigenvector. For the visualization of the dominant motions a pymol script was used which generates depending on the start and end structure arrows to indicate the motion between the carbon α atoms.

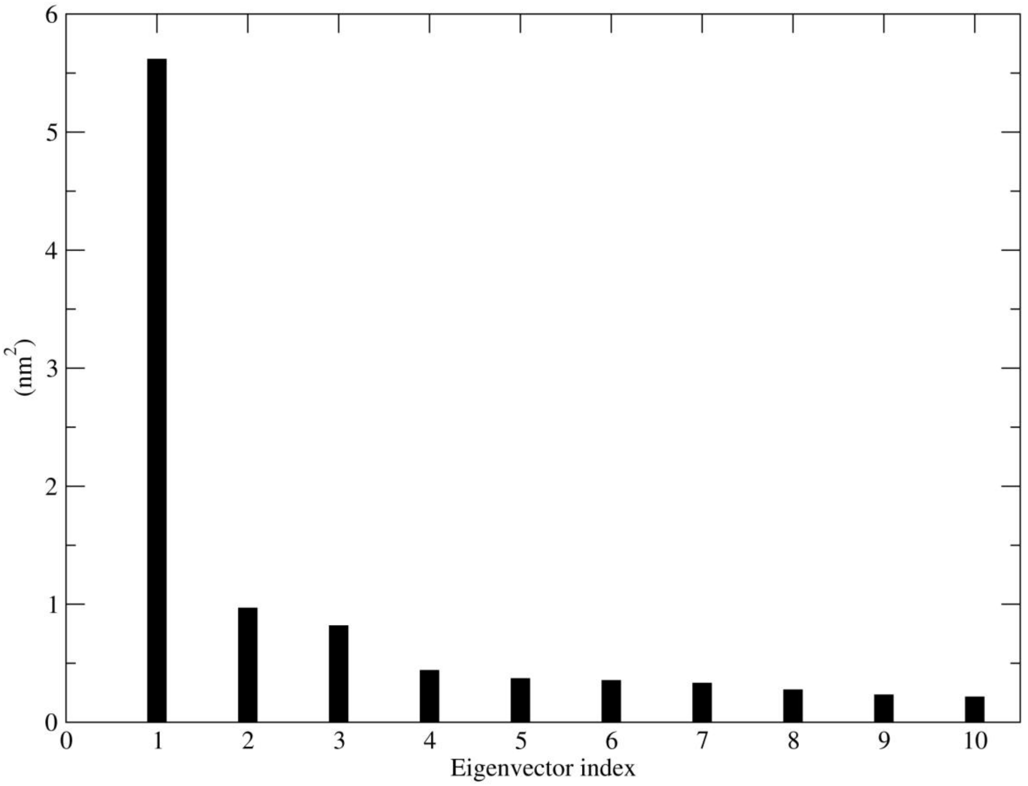

Supplementary Figure 1. Eigenvalues obtained after PCA of the classical MD simulation for the designed hinge peptide.

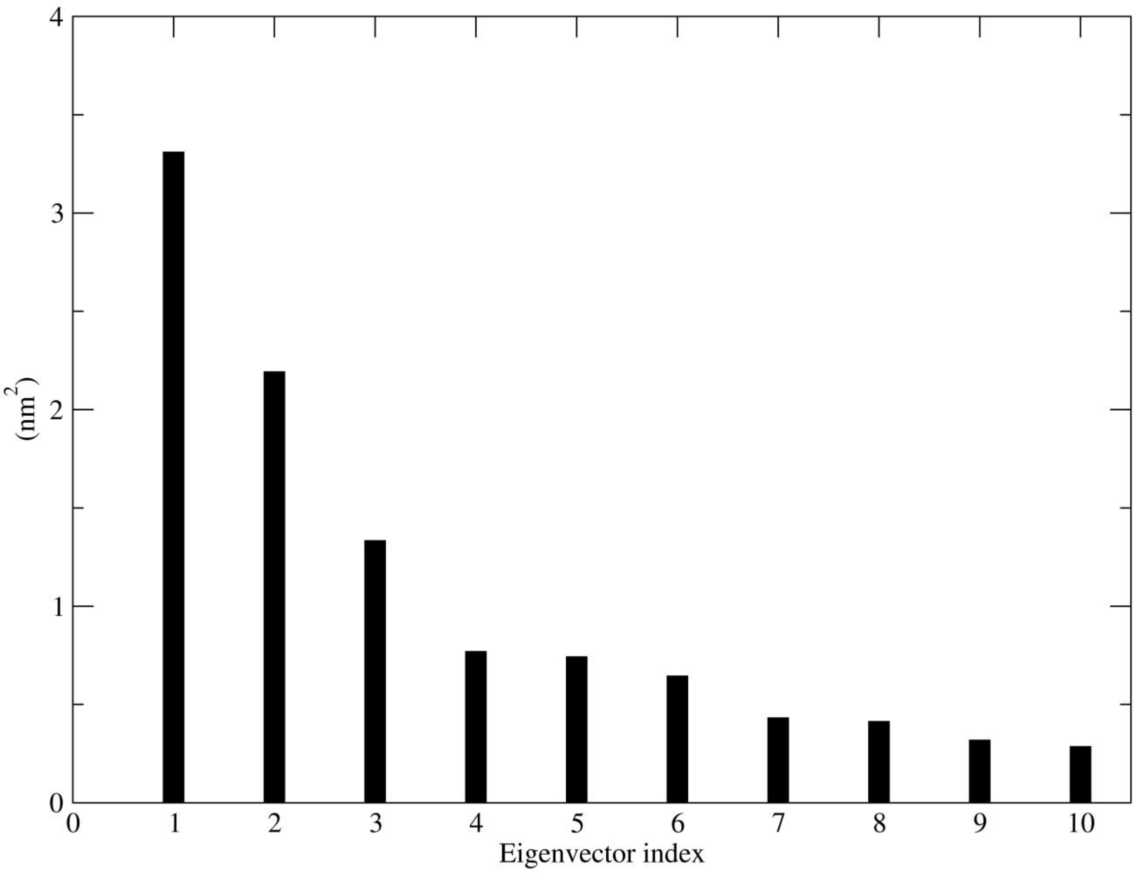

Supplementary Figure 2. Eigenvalues obtained after PCA of the classical MD simulation for the serine-hinge-peptide.

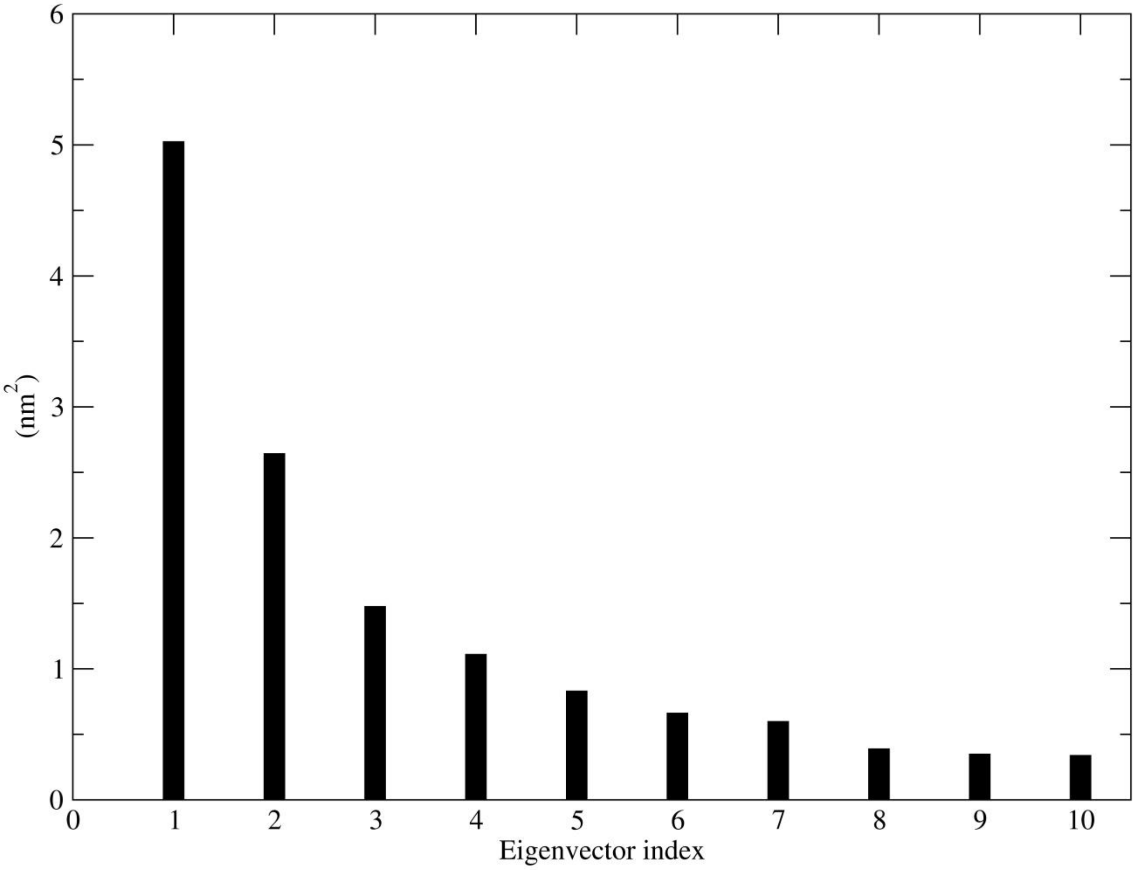

Supplementary Figure 3. Eigenvalues obtained after PCA of the classical MD simulation for the tyrosine-hinge-peptide.

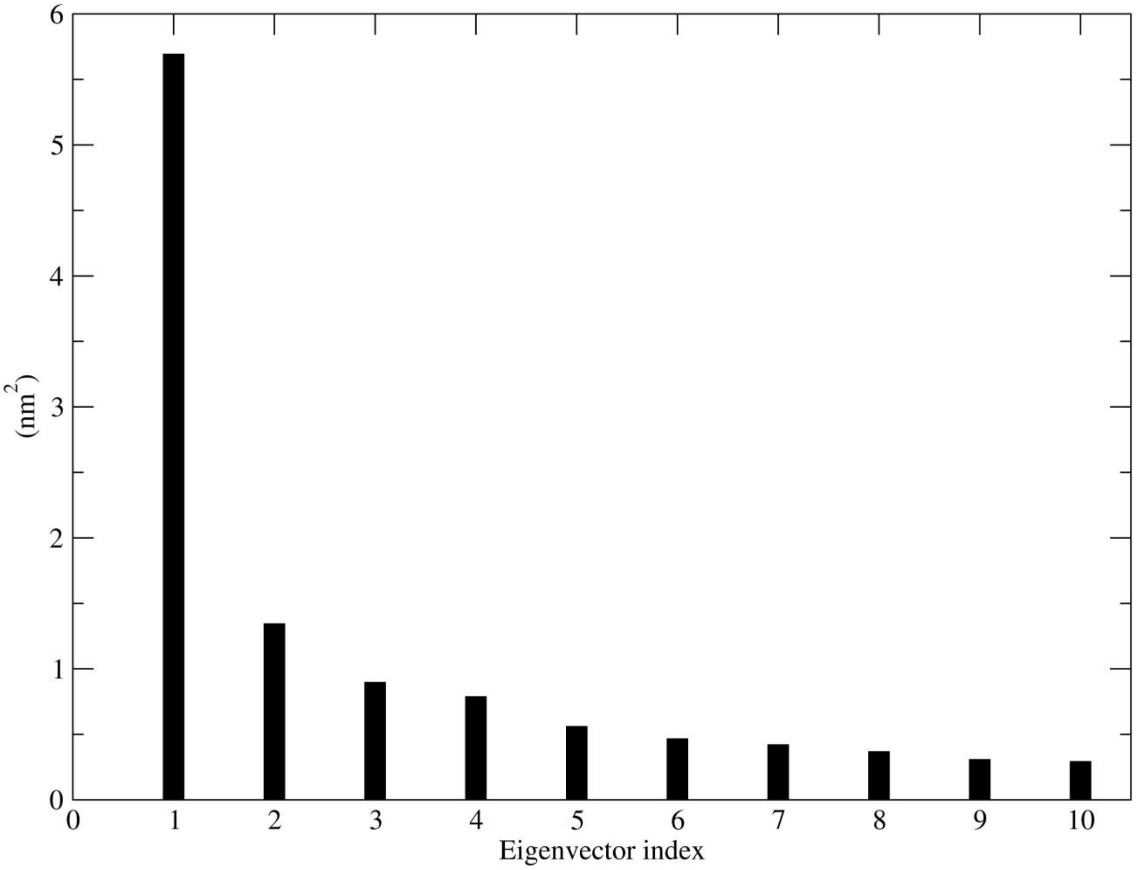

Supplementary Figure 4. Eigenvalues obtained after PCA of the classical MD simulation for the Pro-IgG.

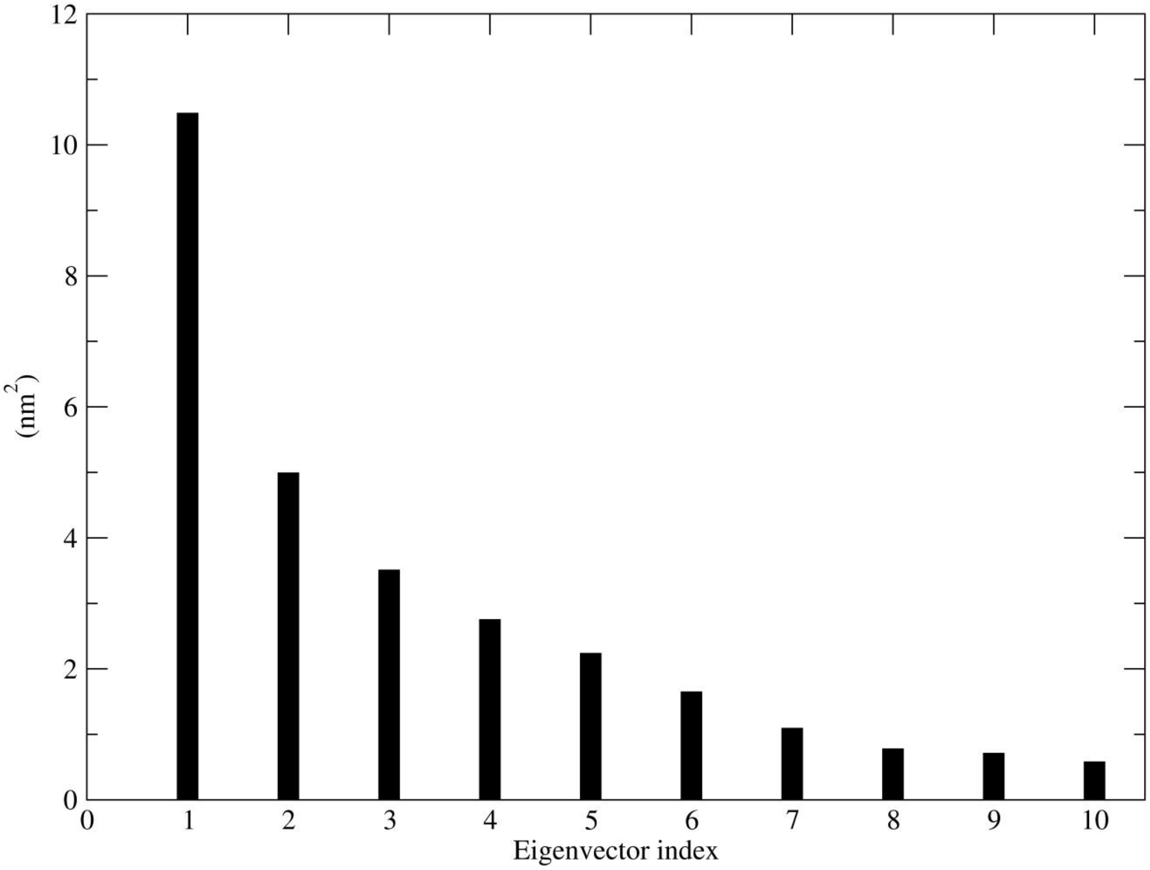

Supplementary Figure 5. Eigenvalues obtained after PCA of the classical MD simulation for the Ala-IgG.

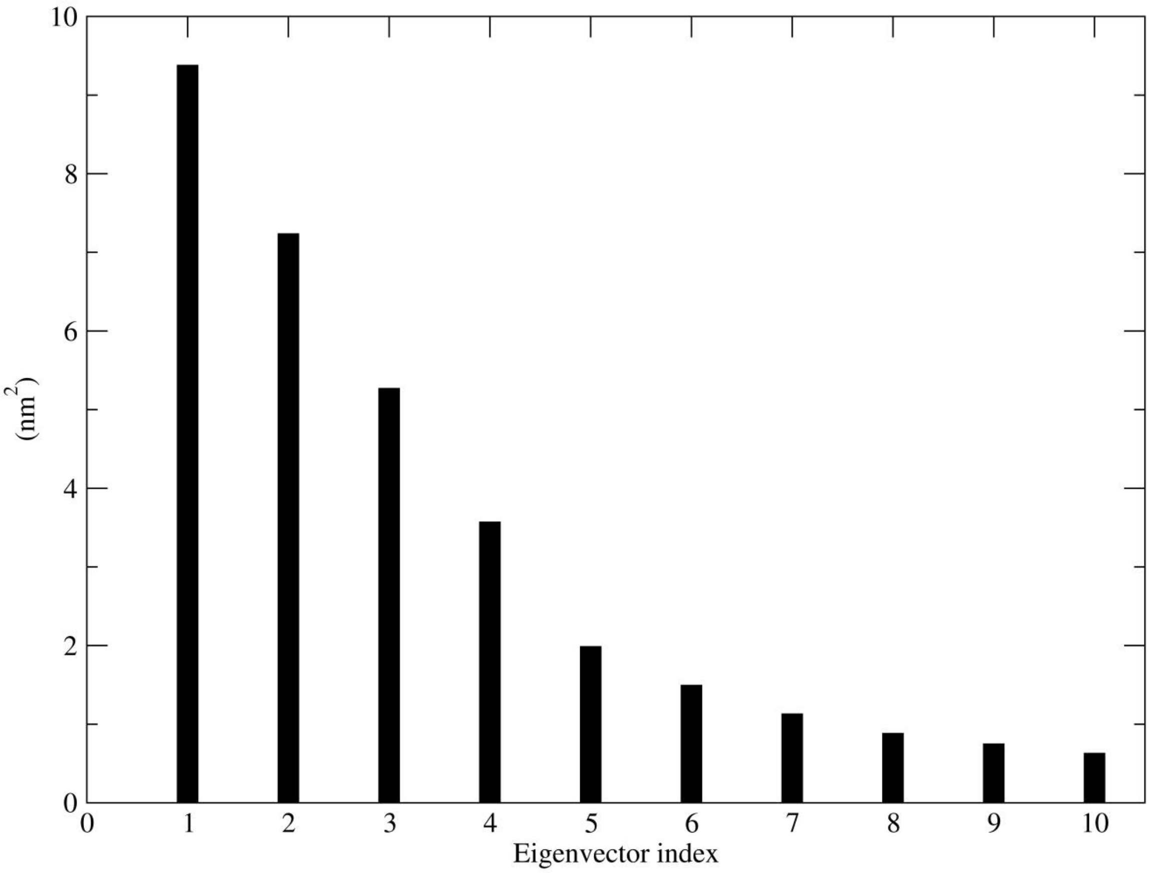

Supplementary Figure 6. Eigenvalues obtained after PCA of the classical MD simulation for the Gly-IgG.

NMR-Structure generation

The NMR-structure of the peptide derivatives were determined by utilizing the Xplor-NIH suite of programs (v. 2.47) which can be obtained for free at (https://nmr.cit.nih.gov/xplor-nih/). For simulated annealing calculations the eefx2 implicit solvent was used. The chains were built separately and joined together by adding the respective disulfide bonds. Distance constraints for the hinge-peptide were extracted from NOESY spectra with a mixing time of 300 ms. The signals were integrated in Topspin and the distance of the tryptophan aromatic protons to another was used as the reference distance. The cross-peaks were thus divided in three categories as weak, medium and strong. Since the system observed a higher flexibility only a small set of 23 well defined NOE-restraints were generated. The analysis of coupling constants revealed preferred side-chain orientation. The dihedral restraint was allowed to deviate up to 15° or 30° in case of the intra-disulfide bonds. This data set proved to be consistent in the deliverance of a structural motif with no NOE-violations above 0.5 Å. In the protocol an extended structure is generated and used for the torsion angle dynamics simulated annealing. For the Pro-IgG the NOESY contacts were obtained

from the literature. (Kessler et al., 1991). For Pro-IgG TCPPCPAP and for the hinge-peptide the sequence CHWECRGCRLVC was used. Both structures were acetylated on the n-terminus. The calculations were performed for 5000 structures which took about 3 days on our system. The simulated annealing protocol in its modified form is supplied in the supp_comput.zip.

MD-Simulations

All simulations were performed using the GROMACS 2018.4 suite patched with plumed 2.4. The GROMACS suite is available for free under the GNU Lesser General Public License at (http://manual.gromacs.org/documentation/) while plumed is available at (https://www.plumed.org/). For input preparation the lowest energy structure originating from the NMR calculations was chosen and processed using the pdb2gmx program set. The n-terminus was acetylated while the c-terminus deprotonated. Histidine and arginine side chains were protonated too. The chains were separated using the "ter" flag. Using the GROMACS suite the system was solvated with ~6000 TIP3P water molecules for the hinge-peptide and ~3000 for the IgG-derivatives in a dodecahedron box and a salt concentration of 0.15M was added to neutralize the system. After evaluation the OPLSAA/M force field was used which is available at (http://zarbi.chem.yale.edu/oplsaam.html). Energy minimization was performed for either 500000 steps or until the maximum force reached a value below 50 kJ/mol/nm using steepest-descent algorithm to remove steric clashes between the peptide and solvent. The next step included a 10 ns long equilibration to allow the solvent to fully surround the peptide. The first equilibration was conducted under a NVT ensemble at 300 K using the modified Berendsen thermostat v-rescale with a coupling time step of 0.1 ps to stabilize the temperature of the system followed by an 10 ns long NPT equilibration to stabilize the pressure using the Berendsen barostat with a coupling time step of 2.0 ps. Particle-Mesh Ewald for the treatment of electrostatic interactions was employed with a short-range cut off of 1.0 nm. For the final unrestrained production run the Parrinello-Rahman barostat was used and the system subjected to a 2200 ns long run at 300K. After the simulation had finished correction to periodic boundary were performed and rotational plus translational motions were removed for easier visualization in the Visual Molecular Dynamics package which can be obtained here (https://www.ks.uiuc.edu/Research/vmd/). Root-Mean-Square Deviation (RMSD) was measured using implemented tools in the GROMACS package. The analytical data were plotted using the grace suite (http://plasma-gate.weizmann.ac.il/Grace/).

Estimation of free energy conversion

One key parameter which controls the quality of a metadynamics simulation is the convergence of the simulation. This can be estimated by the height of the Gaussian deployed during the simulation in combination with the structural fluctuations. The height of the Gaussian slowly declines until 500 ns yet frequent conformational changes can be observed along the CV during the whole simulation frame.

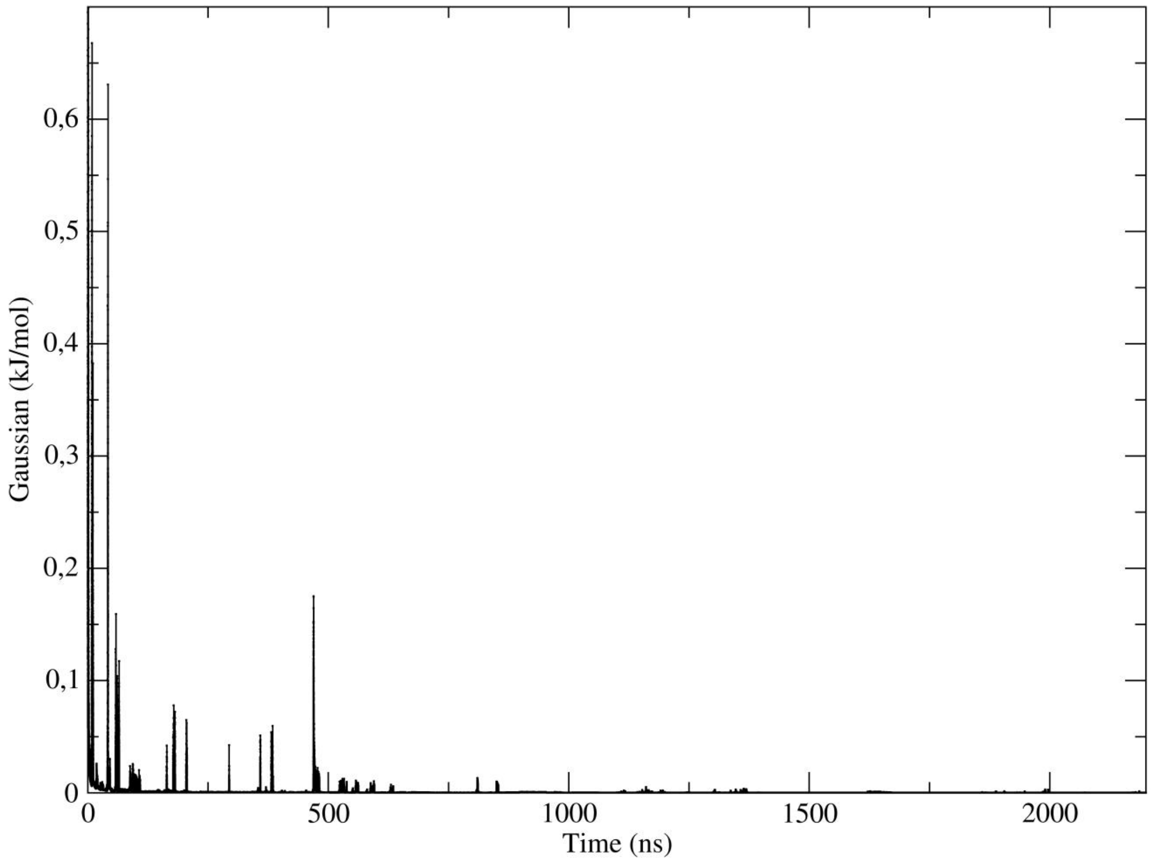

Supplementary Figure 7. Conversion of the Gaussian deposited during the well-tempered metadynamics of Pro-IgG.

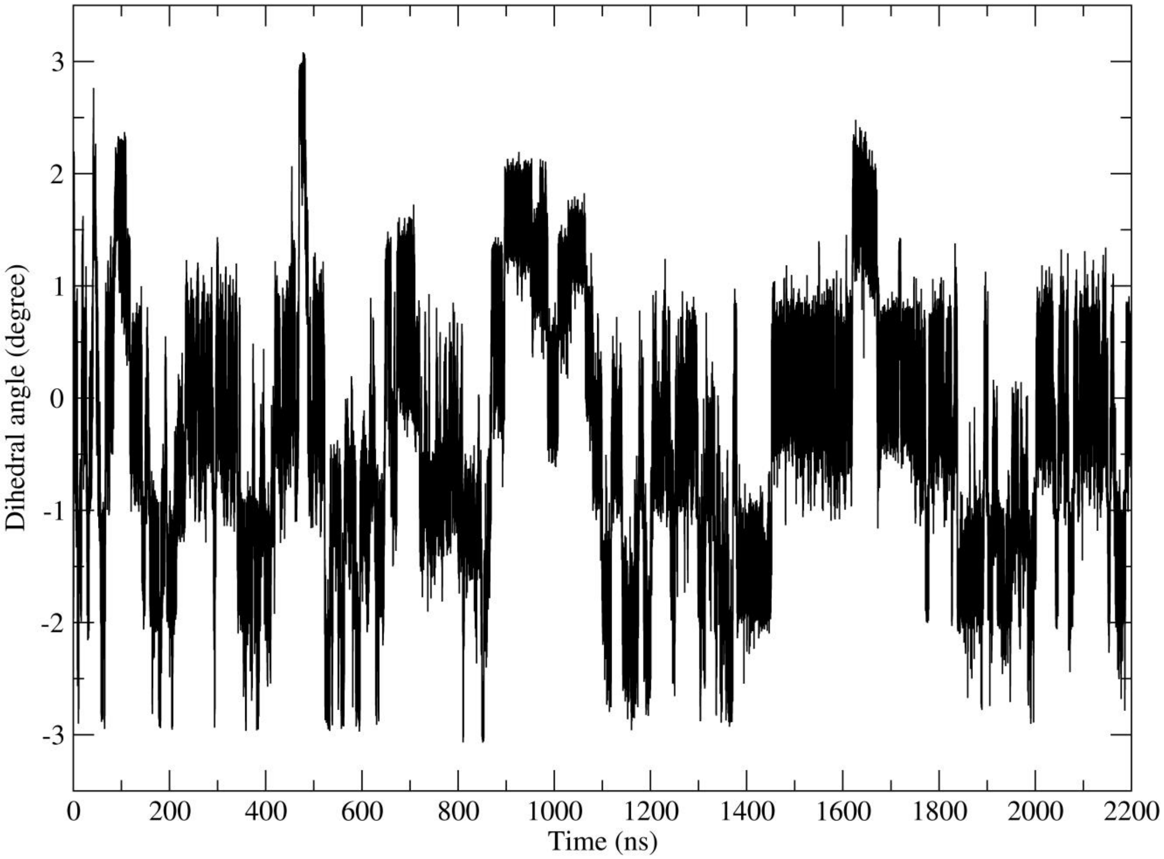

Supplementary Figure 8. Frequent transition of the dihedral angle during the well-tempered metadynamics even after the height of the Gaussian decreased.

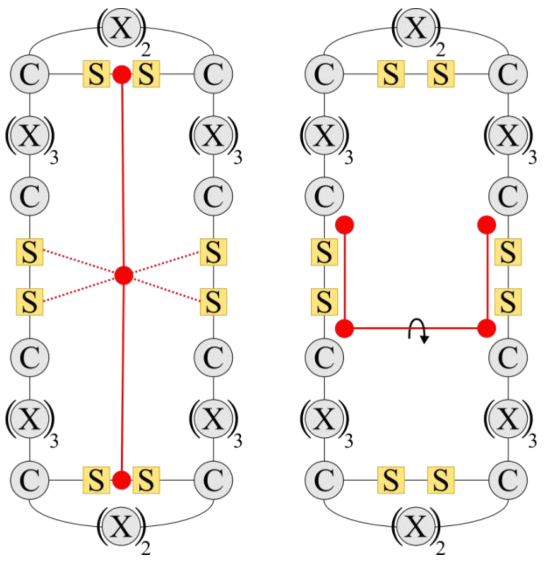

Supplementary Figure 9. Depiction of the sequence of the designed hinge-peptide. On the left the depiction of the opening angle of the hinge spanned by the center between the cysteine residues of the intramolecular disulfides and the center point of the disulfide cluster. On the right the depiction of the torsion of the hinge core built by taking the carbon alpha atoms of the cysteine residues forming the disulfide cluster.

Deposition of trajectories

Every trajectory for standard MD simulation and well-tempered metadynamics is deposited at the OSF depository and are available for free under the following link https://osf.io/jqz3k/?view_only=83558bc693b84753a0eb20d7f7caf0ed. For visualization of the trajectories the structure file xxx.gro has to be loaded into vmd or pymol and the trajectory file has to be appended.

Reference List

Kessler, Horst, Siggi Mronga, Gerhard Müller, Luis Moroder, and Robert Huber. "Conformational Analysis of a IgG1 Hinge Peptide Derivative in Solution Determined by NMR Spectroscopy and Refined by Restrained Molecular Dynamics Simulations." Biopolymers 31, no. 10 (1991): 1189–1204. https://doi.org/10.1002/bip.360311007.
